# Quality of Life and Psychological State in Chinese Breast Cancer Patients Who Received *BRCA1/2* Genetic Testing

**DOI:** 10.1371/journal.pone.0158531

**Published:** 2016-07-18

**Authors:** Jiajia Qiu, Jiaqin Guan, Xiaochen Yang, Jiong Wu, Guangyu Liu, Genhong Di, Canming Chen, Yifeng Hou, Qixia Han, Zhenzhou Shen, Zhimin Shao, Zhen Hu

**Affiliations:** 1 Department of Nursing Administration, Shanghai Cancer Center, Fudan University, Shanghai, China; 2 Department of Breast Surgery, Shanghai Cancer Center, Fudan University, Shanghai, China; 3 Department of Oncology, Shanghai Medical College, Fudan University, Shanghai, China; CNR, ITALY

## Abstract

**Background:**

This study aims to understand the quality of life (QOL) and psychological state (PS) of Chinese breast cancer patients who received BRCA1/2 genetic testing; to examine the psychological changes between BRCA1/2 mutation carriers and non-carriers; and to further explore the psychological experience of BRCA1/2 mutation carriers.

**Methods:**

This study was combined with quantitative and qualitative designs. First, we performed a quantitative investigation using FACT-B (Chinese version) and Irritability, Depression and Anxiety scale (IDA) to assess the QOL and PS in breast cancer patients who received BRCA1/2 genetic testing. Then semi-structured in-depth qualitative interviews among 13 mutation carriers were conducted in hospital.

**Results:**

Results from the quantitative study showed QOL scores were relatively high and the IDA scores were relatively low among the patients, and there was no significant difference in the QOL or IDA scores between non-carriers and carriers. Based on the qualitative analysis, four main themes emerged: (1) Finding the reason for having breast cancer; (2) Negative emotions; (3) Behavioral changes; (4) Lack of information.

**Conclusions:**

The present study showed that QOL and PS are good among the breast cancer patients who received genetic testing. Genetic testing itself does not cause long psychosocial effects. *BRCA1*/*2* mutation carriers may have certain negative emotions at the first stage they knew the testing results and may initiate behavioral and lifestyle changes. The patients with a *BRCA1*/*2* mutation desire knowledge with regard to genetic aspects in mainland China. Professional information and advice can be provided to relieve the patients’ negative emotions when they were informed of gene defect.

## Introduction

Breast cancer is the most common malignancy among women in Shanghai, China, and familial aggregation accounts for approximately 5–10% of breast cancer cases in this region [1]. BRCA1 and BRCA2 are two tumor suppressor genes which have been proven to be breast cancer susceptibility genes. BRCA1/2 mutation carriers have a significantly higher risk of developing breast and ovarian cancer. Patients with breast cancer need to withstand considerable stress from physical illnesses and therapeutic treatment, as well as the psychological, familial, social and other factors. Meanwhile, the result of genetic testing and related information may cause psychological stress to patients or healthy women. Some studies found no difference in the psychological stress between patients suffering from breast cancer and the general population, while other studies found that these patients had higher levels of anxiety throughout the testing process [2]. A survey by Bredart et al [3]showed that short-term potential anxiety was present in BRCA1/2-positive or negative patients. The decision to pursue treatment was relatively easy for patients who have a BRCA1/2 gene defect, while those negative for BRCA1/2 testing struggled with risk management. Schlich-Bakker et al [4] found that patients who underwent genetic counseling had better control over their anxious and repressed emotions. Thus far, limited research has been conducted in this area among Asian populations. Kwong et al [5] interviewed 12 breast cancer patients with a BRCA1/2 mutation who were willing to undergo a contralateral prophylactic mastectomy, mainly to reduce the anxiety about tumor recurrence. The majority of these patients were satisfied with the decision and their psychological burden was reduced.

In China, the popularity of genetic testing remains low and the testing is expensive. Meanwhile, the therapeutic options for BRCA1/2 mutation carriers in China are different from those in other areas. The prophylactic surgeries have seldom been carried out in China. Patients may get the results of testing, but they have no treatment choices. As a consequence, little is known about the psychological feelings and psychosocial conditions of Chinese breast cancer patients who have a BRCA1 or BRCA2 mutation, leading to a significant deficiency in nursing care and intervention. The objectives of the present study are to understand the quality of life (QOL) and psychological state (PS) of breast cancer patients who received BRCA1/2 genetic testing; to examine the psychological changes between BRCA1/2 mutation carriers and non- carriers; and to further explore the psychological experience of BRCA1/2 mutation carriers. The results will provide a reference for the pattern of nursing care among breast cancer patients who have a BRCA1/2 mutation, to develop effective nursing interventions of these patients.

## Methods

### Ethical approval

This study was approved by the Scientific and Ethical Committee of the Shanghai Cancer Center, Fudan University. Written informed consent (S1 File) was obtained from all participants before data collection. The individuals discussed in this manuscript have given written informed consent to publish these details.

### Design and Data Collection

This study was designed to use both quantitative and qualitative methods. First, we used questionnaires to assess the whole QOL and PS in patients who received BRCA1/2 genetic testing. After the preliminary investigation, we planned to obtain in-depth insights into psychological experience in BRCA1/2 mutation carriers at their first stage they knew the testing results.

#### Quantitative study

A total of 99 breast cancer patients from families of cancer patients regardless of cancer types were screened. These patients entered Professor Zhen Hu’s study entitled “Identification of a Comprehensive Spectrum of Genetic Factors for Hereditary Breast Cancer in a Chinese Population by Next-Generation Sequencing” in our cancer center before [6]. In their study, the breast cancer patients were required to meet one of the following inclusion criteria [6]:1)Age younger than or equal to 35 years with at least one other blood relative suffering from any type of cancer; 2)Age older than 35 and younger than or equal to 50 years with 2 blood relatives in the same lineage suffering from any type of cancer; 3)Age older than 50 years with 3 blood relatives in the same lineage suffering from any type of cancer. From May to October 2014, we connected with these 99 breast cancer patients. To be eligible for our study, the breast cancer patients should show no serious complications; had no past or present history of mental illness or consciousness disturbance, and all were willing to participate in the survey. Among them, twenty-three patients refused to accept our quantitative investigation and seventy-six patients entered our study.

We used questionnaires to assess the QOL and PS in breast cancer patients undergoing BRCA1/2 gene testing including FACT-B (Chinese version) [7] and Irritability, Depression and Anxiety scale (IDA) [8].

FACT-B was composed of a general module (FACT-G) that measures general QOL in cancer patients and a breast cancer-specific module [9]. FACT-G was composed of 27 items, which were divided into 4 parts. The breast cancer-specific module contained 9 items. A higher score indicated better QOL. By explanatory translation, retroversion and cultural adjustment, the scale was translated in Chinese in 1998 and it’s been proved to have a good reliability and validity in Chinese population [7,10]. The Cronbach α of each subscale were 0.84, 0.84, 0.79, 0.83 and 0.61 [10]. The correlation coefficients between all subscales were all above 0.65 [10].

IDA was developed by Saith RP and is composed of 18 items, which were designed to assess irritability, depression and anxiety respectively [8]. A lower score indicated better psychological state. IDA was translated into Chinese by Yuan et al [11] and was proved to have good reliability and validity. The Cronbach α of each subscale ranged from 0.419 to 0.769 [11]. The correlation coefficients between all subscales of IDA ranged from 0.400 to 0.776 [11].

Researchers contacted patients through telephone, explained details of the study to each patient, and asked for each patient’s informed consent to participate in the study. If consent was given, patients were invited to the hospital to participate in the quantitative survey.

#### Qualitative study

In Professor Zhen Hu’s study, eighteen patients were found to carry BRCA1/2 germline mutations [6]. Following the quantitative survey, the targets of qualitative study were selected by purposive sampling. The inclusion criteria should show no past or present history of mental illness or consciousness disturbance; were mandarin-speaking; and were willing to be interviewed. The exclusion criteria were severe cardiovascular disease and brain dysfunction. From October 2014 to January 2015, researchers contacted these eighteen patients through telephone or at their following-up visit, explained the study to each patient in detail and asked for their informed consent to participate in the study. Among the eighteen patients, five patients refused enrollment and thirteen BRCA1/2 mutation carriers consented to participate in qualitative interview. Then participants were scheduled to be interviewed at their next clinic visit. Informed consents were obtained before each interview.

Semi-structured interview was designed. At the beginning of each interview, the researchers explained the purpose of the study to the participants. A semi-structured interview guide helped the interviewer to focus on important topics during the whole process. Questions were developed based on existing literature review and clinical experience. Key topics included: understanding of genetic testing (the reason to accept the genetic testing); emotional and behavioral status after knowing about the results of genetic testing; and the information or other demands for genetic counseling.

To ensure the participants’ privacy, the principal researcher conducted all interviews in a quiet and comfortable room in the Center. In a semi-structured interview, participants were asked for questions verbatim in the same order. With the participants’ permission, the principal researcher tape-recorded in the interview and transcribed them verbatim. Each interview lasted between 30 and 45 minutes. At the end of each interview, the participant was given a prepaid public transportation card that worth RMB100 (approximately 16 US dollars) for participating in the study. Demographic and treatment data were also collected via the personal information form.

### Data Analysis

#### Quantitative study

Data were sorted and analyzed using SPSS 18.0. The mean, standard deviation and percentage were used to describe the basic data of the patients and their QOL and IDA scores. T-tests were performed to compare the QOL and IDA scores between BRCA1/2 mutation carriers and non-carriers.

#### Qualitative study

Content analysis, as described by Colaizzi [12], was used in data analysis. The recorded interviews were transcribed verbatim and later compared with the audio recordings to ensure the accuracy of the transcripts. The researchers continuously read the data, highlighted important points or wrote comments in the margins. The transcripts were reviewed line by line, The raw data were grouped after repeated readings. Codes were developed based on the original terms used by participants by 2 researchers. The significant statements were checked and consulted within the research team. Then the meanings were created from the clustered codes and major themes were attached by the principal researcher. To ensure the authenticity of the contents, the participants were invited to review the summary of our findings at their following-up visit, the principal researcher contacted them through telephone in advance. Participants were asked how the themes developed by researchers compared with their own perspectives, to ensure that an accurate representation of the participants’ perspectives had been sustained.

In this manuscript, all quotations from participants are indicated in italics. Aliases (Ms A to M) were used for the participants.

## Results

### Findings from the Quantitative Study

The subjects had an average age of 45.76 years (range 23–73 years). The majority of the patients were married (86.8%) and had children (77.6%). A small proportion (22.4%) had religious beliefs. Regarding the degree of education, college/university and high school graduates accounted for 60.5% and 18.4% respectively. The average disease course lasted 52.97 months (range 25–173 months), and the average time since the patients knew the genetic testing results until they took part in our study was nearly one year (range 10–13 months).

1. QOL and PS scores of breast cancer patients who received genetic testing
  The QOL scores were relatively high, and the IDA scores were relatively low among the subjects. (Tables 1 and 2)
2. Comparison of QOL and PS scores between *BRCA1*/*2* mutation carriers and non-carriers
  There was no significant difference in the QOL or IDA scores between the two groups of breast cancer patients. (Tables 3 and 4)

**Table 1:** QOL Scores of breast cancer patients who received genetic testing (FACT-B) (N = 76).

Item	Score(x±s)	Score range	Scores in Chinese BC Patients*^1^(x±s)
PWB	23.95±3.64	0–28	20.70±4.83
SWB	20.01±8.34	0–28	19.76±5.24
EWB	18.21±3.78	0–24	16.23±4.93
FWB	18.87±6.69	0–28	14.61±5.86
ACB	22.93±4.48	0–36	24.79±4.45
TOTAL	103.97±17.92	0–144	94.81±18.87

*QOL*, Quality Of Life; *BC*, Breast Cancer

*^1^ Subjects were tested when FACT-B was translated into Chinese.

**Table 2:** PS Scores of breast cancer patients who received genetic testing (IDA) (N = 76).

Item	Score(x±s)	Score range	Scores in Chinese Patients with depression*^2^(x±s)
Depression	4.42±2.54	0–15	8.78±3.56
Anxiety	5.25±2.53	0–15	8.45±2.96
Inward Irritability	2.09±2.08	0–12	5.09±2.88
Outward Irritability	3.41±2.18	0–12	4.23±2.80

*PS*, Psychological State

*^2^ Subjects were tested when IDA was translated into Chinese.

**Table 3:** Comparison of QOL scores between *BRCA1*/*2* mutation carriers and non-carriers (FACT-B) (N = 76).

Item	mutation carriers(x±s) (n1 = 17)	mutation non-carriers(x±s) (n2 = 59)	P
PWB	23.00±3.41	24.22±3.69	0.226
SWB	21.24±6.04	19.66±8.90	0.496
EWB	17.71±3.67	18.36±3.83	0.536
FWB	18.47±5.94	18.98±6.93	0.783
ACB	21.71±4.43	23.29±4.46	0.201
TOTAL	102.12±16.16	104.51±18.49	0.631

*QOL*, Quality Of Life

**Table 4:** Comparison of PS scores between *BRCA1*/*2* mutation carriers and non-carriers (IDA) (N = 76).

Item	mutation carriers(x±s) (n1 = 17)	mutation non-carriers(x±s)(n2 = 59)	P
Depression	4.06±2.19	4.53±2.64	0.508
Anxiety	5.65±2.50	5.14±2.54	0.466
Inward Irritability	2.18±1.85	2.07±2.16	0.851
Outward Irritability	3.41±2.43	3.41±2.13	0.993

*PS*, Psychological State

### Findings from the Qualitative Study

Thirteen women with breast cancer participated in this study. The subjects had an average age of 43.9 years (range 34–65 years). The average disease course was 52.2 months (range 28–120 months). There was 1 unmarried patient and 2 nulliparous patients. 9 patients held a college or Bachelor’s degree and 9 patients underwent modified radical surgery. All patients received chemotherapy and 9 received radiotherapy. (Table 5)

**Table 5:** Basic characteristics of participants in in-depth interviews (N = 13).

Name (anonymous)	Age	Educational Level	Marital Status	Children	Time since Diagnosis(Month)	Type of Surgery	Adjuvant Therapy
Ms. A	37	College	Married	1	28	Mastectomy	CT
Ms. B	41	College	Married	1	36	B+S	CT+RT
Ms. C	39	Junior College	Unmarried	0	28	B+S	CT
Ms. D	54	High School	Married	1	44	Mastectomy	CT+RT
Ms. E	36	College	Married	0	29	B+S	CT+RT
Ms. F	54	College	Married	1	102	Mastectomy	CT+RT+ET
Ms. G	47	Junior School	Married	1	88	Mastectomy	CT
Ms. H	48	High School	Married	1	29	BCS	CT+RT+ET
Ms. I	65	College	Married	1	120	Mastectomy	CT+RT+ET
Ms. J	36	College	Married	1	39	Mastectomy	CT+RT
Ms. K	40	High School	Married	1	31	Mastectomy	CT+ET
Ms. L	41	Junior College	Married	1	55	Mastectomy	CT+RT+ET
Ms. M	34	Junior College	Married	1	50	Mastectomy	CT+RT+ET

*B+S*, Breast-Conserving Surgery +Sentinel Biopsy; *BCS*, Breast-Conserving Surgery; *CT*, Chemotherapy; *RT*, Radiotherapy; *ET*, Endocrine Therapy; *HT*, Herceptin Therapy;

The following main themes were extracted based on the results of qualitative analysis: (1) Finding the reason for having breast cancer; (2) Negative emotions; (3) Behavioral changes; (4) Lack of information.

#### Finding the reason for having breast cancer

Given the inclusion criteria specifying patients with a family history of breast cancer, the subjects had a certain subconscious suspicion regarding the result of the genetic testing. “*My mother has already been proven to have this illness*. *Well*, *since I have the same illness*, *it is certainly due to the problem of the gene*. *… The reason why I am ill is found*, *and I think it is because of the powerful gene” (Ms*. *E*, *36-year-old*, *unmarried and nulliparous)*; *“How to explain the early illness when I am so young*? *It is because of my gene defect*. *It is inevitable*. *… I finally found a reason” (Ms*. *L*, *41-year-old*, *married and parous)*. Based on these suspicions, the patients were not very surprised to learn of the positive result of genetic testing and seemingly calmly accepted the “gene defect”. *“If we had no family history*, *I must be very depressed about having this illness*. *… Now that the gene is so powerful in my family history*, *there is no way to deal with it” (Ms*. *D*, *54-year-old*, *married and parous)*; *“I have no particular reaction but feel clear that I am ill so early only because of the gene mutation” (Ms*. *J*, *35-year-old*, *married and parous)*; *“I think this bad gene is the cause of my illness*. *… Other people may have more fear if they did not find the cause” (Ms*. *M*, *34-year-old*, *married and parous)*.

#### Negative emotions

Having cancer is a formidable stressful event, while the positive result of genetic testing brings more psychological distress to the patients at the first stage they knew the testing results. Meanwhile, the treatment options and prophylactic methods are currently limited, making the patients feel helpless.

1. Fear: The literal meaning of “gene defect” and “genetic problem” is sufficient to make the patients upset.
  - For oneself: The physical and psychological fatigue as well as the difficulties and hardships in response to the disease made the patients particularly concerned about the disease “occurring one more time”. Meanwhile, they would connect all of the symptoms to the previous disease condition. *“My greatest fear is contralateral breast recurrence*. … *What I am most worried about is to have chemotherapy once again—This is what makes me most frightened*. …*Nowadays*, *I will feel upset as long as my period is slightly unusual*, *mainly because of the huge psychological stress*. … *I know I am at high risk for contralateral breast cancer*. *The situation may not be so serious if I had no gene defect” (Ms*. *C*, *39-year-old*, *married and nulliparous)*.
  - For children: Among the subjects interviewed, 11 patients had children. The genetic predisposition thus made them especially worry about their offspring. *“I am afraid that he (the son) will have the same misfortune (sobbing)*. … *I am thinking if my boy has to follow the same destiny*, *then I want him to live a comfortable life*. *Although the gene may not develop symptoms*, *living happily will definitely improve the quality of life (crying)” (Ms*. *A*, *37-year-old*, *married and parous)*; *“It is about my daughter*. … *I am so worried that she would follow my footsteps sooner or later (eyes moistened)” (Ms*. *G*, *47-year-old*, *married and parous)*. Moreover, the patients carried the sense of guilt toward their children. *“Actually*, *I was most worried about my kid*. … *What if my kid also has inherited this gene*, *and the congenital deficiency leads to certain consequences*?*” (Ms*. *J*, *35-year-old*, *married and parous)*.
2. Despair: The patients fell into deep despair due to scattered knowledge of the disease and the lack of treatment options and prophylactic measures in China. *“The key problem is that even though I am aware of it*, *there is still no cure*. *I think it is good to learn of it if there is a way to cure it*. *Now I feel very desperate" (Ms*. *A*, *37-year-old*, *married and parous)*; *“Can I only stand to see it recur on the right side*? *What if it metastasizes*? *… Maybe I will not feel so much stress if I have the contralateral breast removed as well” (Ms*. *C*, *39-year-old*, *married and nulliparous)*.

#### Behavioral changes

Having cancer was the first warning for the patients and their family, while the gene mutation was the second warning for the patients. The patients paid attention to a healthy diet, good lifestyle and timely follow-up. They also reminded family members to be vigilant and brought about behavioral changes in the whole family through their own effort.

1. Change oneself: With the alerts of having cancer and the gene mutation, the patients started to alter their actions according to the implication of these warnings. *“Once I learned of my gene defect*, *I would not work like before with high strength and much pressure*. *Then*, *I would not go to restaurants for food…*. *I should change a lot of my lifestyle that may hurt myself because of the gene defect” (Ms*. *C*, *39-year-old*, *married and nulliparous)*.
2. Urge family members to change behavior, especially children: Although the patients thought that there was no way to change the current situation, they still constantly reminded their family members to change their behavior, especially their children. *“She (the daughter) now also feels the need to take care of her health…*. *I have been saying that she must keep this ideal in her mind and go to check it after turning 30 years old” (Ms*. *G*, *47*-*year-old*, *married and parous)*; *“This illness is a warning to remind my daughter of relevant examinations” (Ms*. *I*, *65*-*year-old*, *married and parous)*.

#### Lack of information

1. State of “ignorance” of relevant information: All patients noted their ignorance of the treatment for the disease. None was aware of relevant information or the latest developments in China and overseas, either obtained relevant knowledge from the medical staff. *“Genetic testing is sophisticated anyway*. *I know little about it*, *but it sounds very scientific" (Ms*. *B*, *41*-*year-old*, *married and parous)*; *“I often listen to the radio*, *so I know this disease is very likely to recur*. *But I know little about the treatment*. *… I do not understand it either” (Ms*. *F*, *54-year-old*, *married and parous)*.
2. Demands in specialized knowledge: There is a huge gap between Chinese and foreign patients in the pathway to obtain gene-related specialized knowledge and the timeliness of obtaining this knowledge. All subjects interviewed in this study were aware of the demands in specialized information. *“I certainly want myself to be treated*. .… *For my son*, *I have no clue where to start*. .… *If the testing result is good*, *then the trouble will be gone; if the result is bad*, *maybe I will lower my requirements on him*. .… *I am very eager to learn these*, *just like others” (Ms*. *A*, *37-year-old*, *married and parous)*; *“After all*, *I want to learn the latest information on this illness*. … *If I had tested blood for this gene when I first had breast cancer (in 2007)*, *and if the result was positive*, *I would definitely have had the ovaries removed*. *In that case*, *I would not suffer from ovarian cancer*. *The result of genetic testing more or less has influence on my decision” (Ms*. *G*, *47-year-old*, *married and parous)*.

## Discussion

The present study was the first to investigate the PS and QOL of breast cancer patients who received *BRCA1*/*2* genetic testing and to investigate the psychological experience of *BRCA1*/*2* mutation carriers in mainland China. Currently, genetic testing and genetic counseling are still not popular and prophylactic treatment has not been carried out in China. Therefore, the psychological reaction of breast cancer patients to genetic testing was unknown in mainland China.

Compared to the population which FACT-B(Chinese-version) was tested, the total QOL score was ranked above average, and the individual scores for the physiological, social-family, emotional, and functional conditions and for the additional concerns were relatively high, indicating a good QOL in our patients of interest who received BRCA1/2 genetic testing. This result is consistent with a previous study of the QOL in community patients by Xue et al [13], indicating that there was no difference in the QOL between general breast cancer patients and those who already took genetic testing. In our study, the average disease course was 52.97 months among the patients. Because the period of convalescence after treatment was relatively long, the patients restored satisfying physiological functions and satisfying psychological conditions. Moreover, the total QOL score and individual dimensions were not different between *BRCA1*/*2* mutation carriers and non-carriers among the patients. Smith et al [14] indicated that QOL differences between individuals with and without the gene defect only occurred at 3 months but disappeared at 6 months. In our study, the average time since the patients knew the genetic testing results until they took part in the study was one year. When the patients are informed of the gene defect, a short-term decline in physical and psychological performances might occur. However, this news would not affect the long-term QOL of the patients. In another study by Hooker et al [15], breast cancer patients with a gene defect were satisfied with the genetic testing and their QOL was also good.

While genetic testing provides a certain amount of information to high-risk patients, it may also cause psychological stress. In our study, the patients who received genetic testing exhibited no obvious anxiety, depression or irritability and there was no difference in such negative emotional reactions between patients with and without the gene mutation. One of the reasons is that gene mutation carriers might be worried and anxious when they are informed of the result, but these feelings gradually disappear in approximately one year [16]. Bredart et al [3]investigated 243 breast cancer patients, 11% of whom had a gene defect. It was found that short-term potential anxiety existed in *BRCA1/2* mutation-positive or negative patients, while the long-term effect was limited. It is consistent with our quantitative finding- the average time since our patients knew the genetic testing results until they took part in our study was one year. The other reason is that the Chinese patients know little about the genetic testing and hardly have access to the relevant knowledge. Such ignorance and neglect, instead, leads to their emotional stability.

Although there was no significant QOL and psychological difference between patients with or without mutation, literature reviews revealed short-term potential decline in psychological function when patients were informed of gene defect. So after quantitative investigations, we tried to deeply understand psychological experience in mutation carriers when they were informed of gene defect. In our qualitative interview, the BRCA1/2 carriers really show some negative emotion at the first stage they knew the results of genetic testing. The patients in our study, especially the younger ones, said that the positive result of genetic testing implied that they might have the disease. Given the inclusion criteria for breast cancer patients with a family history, the majority of the patients had subconsciously regarded “mutation” as an inevitable result based on the situation of familial cancer. The patients just confirmed the reason for their illness and accepted the result. However, “Carrying the mutation” is indeed not good news [17], it at least provides a solid reason for having cancer [18].*BRCA1*/*2* mutation carriers may worry about the recurrence of cancer and do not want to go through the painful process once again [17]. The awareness of the gene defect makes the patients distressed when they are informed of their results.

Breast cancer patients choose genetic testing because it can estimate the risk of having breast cancer again and help make the decision to pursue prophylactic treatment. Uyei et al [19] investigated 554 women undergoing genetic testing, among whom 132 had a gene mutation. In these gene mutation carriers, 12% chose prophylactic radical surgery, 12% chose prophylactic bilateral oophorectomy plus salpingectomy, 24% chose both of these surgeries, and the remaining patients chose follow-up monitoring. In China, the treatment modalities for breast cancer patients with a mutation are different from those overseas. Few prophylactic treatments and no regular genetic counseling have been carried out, so Chinese patients lack control of and choices for risk management. There are fewer options for Chinese patients, and except for close follow-up, other treatments have not been implemented appropriately in China. Foreign patients can be kept informed of specialized knowledge about genetic testing, treatment and prophylaxis of breast cancer. They can actively discuss with their doctor the pros and cons of various treatment options and the choice of preventive measures. But in China, the patients feel helpless and powerless because they know the result but are incapable of doing anything. They are unaware of how to make the right decision, and this is an important cause of negative emotions in *BRCA1*/*2* mutation carriers, especially at the very beginning they got their results. Almost all of the subjects interviewed in this study felt distressed due to the lack of information after learning of their gene defect. These patients felt that genetic testing had a certain role in directing and reminding them, but “being incapable of doing anything” made them distressed and despair. The patients desire the latest information in specialized fields to cope with the problem affecting themselves and their family. Research indicates that the risk of gene-related cancer and the patients’ uncertainty regarding risk management and decision making can be reduced by the effective use of specialized information systems [20]. This strategy is even less common in specialized areas in China, and none of our patients took seriously the relevant knowledge or advice from the medical staff.

Genetic testing itself may not cause long psychosocial effect. Knowing about the results of genetic testing at first stage may cause some negative emotion and the patients need informational and psychological support. And they could gain an improved awareness of specialized information after genetic counseling [21], which can help them to make the right and appropriate decisions, without increasing the psychological stress or reducing the QOL of the patients. Hence, there is a great need to carry out genetic counseling for high-risk patients or women in mainland China, providing specialized support and services for a large number of Chinese patients with breast cancer.

### Study limitations

The present study has some limitations. The survey was conducted one year after patients learned the results of genetic testing. The initial PS of the patients when learning the results remains unknown. The short- and long-term PS and QOL of breast cancer patients who received genetic testing will be the focus of our future research. Meanwhile, our study on genetic counseling is still in its initial stage. The content and pattern of genetic counseling will be further improved in future research, to provide specialized information and support to breast cancer patients who received genetic testing.

## Conclusions

QOL and PS are good among the breast cancer patients who received genetic testing. Genetic testing itself does not cause long psychosocial effects. *BRCA1*/*2* mutation carriers may have certain negative emotions at the first stage they knew the testing results and may initiate behavioral and lifestyle changes. The patients with a *BRCA1*/*2* mutation desire knowledge with regard to genetic aspects in mainland China. Professional information and advice can be provided to relieve the patients’ negative emotions when they were informed of gene defect.

## Supporting Information

S1 File Informed consent. (PDF)
