# Supplementary material for: Quality of Life and Psychological State in Chinese Breast Cancer Patients Who Received BRCA1/2 Genetic Testing
Source: PLoS One. 2016 Jul 18;11(7):e0158531. doi: 10.1371/journal.pone.0158531 (PMC4948873; doi:10.1371/journal.pone.0158531)
Supplement: S1 File — (PDF) [file pone.0158531.s001.pdf]

知情同意书

BRCA1/2 基因突变乳腺癌患者的心理体验

1、____________________ 您好！

我们是复旦大学附属肿瘤医院（简称肿瘤医院）的研究人员，现进行 BRCA1/2 基因突变乳腺癌患者的心理体验的质性研究。这项研究是自愿参加的，研究的实施将遵守相关的法律和法规。本知情同意书解释了研究的目的和内容。请仔细阅读下文，在您对本研究有足够的了解后决定是否参加。

2、<u>研究目的</u>

主要目的是了解中国 BRCA1 或 BRCA2 基因突变乳腺癌患者的身心需求，探讨中国 BRCA1 或 BRCA2 基因突变乳腺癌患者的心理情感体验。

3、<u>研究内容</u>

本次研究以访谈的形式完成，我们会邀请您参加访谈，并录音和记录下相关内容。

4、<u>可能的受益</u>

此次调查的结果将有助于了解中国 BRCA1 或 BRCA2 基因突变乳腺癌患者的身心需求和情感体验，从而对设计针对此类乳腺癌患者的随访模式有所帮助，可以使以后的患者从此项研究中获益，但此项研究中您可能不会受益。

5、<u>可能出现的风险和不良反应</u>

在访谈中提及的某些问题可能会比较敏感，您可以拒绝回答任何使您感到尴尬的问题。

6、研究程序的改变

您可以选择不参加此项研究，这不会对您在肿瘤医院的治疗和护理产生影响。

<u>对于患者医疗信息资料的使用授权：</u>

1、研究期间我们将收集您的信息资料（包括您的病史、治疗、调查问卷）。您的医生、研究者可以查询这些资料。

您有权在本研究的所有患者完成研究之后查询并复印您的信息资料。

2、您在本文件中同意我们使用您研究中的资料。

3、如果您在拒绝授权我们使用您的信息资料，您将不能参加这项调查。

4、我们将保护您的私人隐私信息。

患者已经理解并同意以下声明：

1、 我的参与是自愿的。

2、我可以向研究人员（裘佳佳，联系方式 64175590-88701）询问与研究相关的任何问题，向伦理委员会询问被调查者的权利等问题。

3、我可以在任何时间退出该研究，不会因此遭受损失或者给治疗带来不利影响，但退出前我应该向我的研究人员声明。

4、肿瘤医院会采取合适的措施来保护我个人医疗信息资料的安全性。研究者可以查阅我的资料收集数据并确保这项研究的安全性和正确性。

5、我将获得此份知情同意书的副本。

同意

我已经阅读这份知情同意书并自主同意参与这项调查。

患者签字：　　　　　　　　　　　　日期

我已经将研究内容向患者作了解释，并且已经得到他（她）对于知情同意的理解。

调查者签字：　　　　　　　　　　　　日期
